# Gestational exposure to an epidemiologically defined mixture of phthalates leads to gonadal dysfunction in mouse offspring of both sexes

**DOI:** 10.1038/s41598-019-42377-6

**Published:** 2019-04-23

**Authors:** Anastasia Repouskou, Emily Panagiotidou, Lydia Panagopoulou, Pernilla Larsdotter Bisting, Astrud R. Tuck, Marcus O. D. Sjödin, Johan Lindberg, Evangelos Bozas, Joëlle Rüegg, Chris Gennings, Carl-Gustaf Bornehag, Pauliina Damdimopoulou, Antonios Stamatakis, Efthymia Kitraki

**Affiliations:** 1 Laboratory of Basic Sciences, Faculty of Dentistry, School of Health Sciences, National and Kapodistrian University of Athens (NKUA), Athens, Greece; 2 Biology-Biochemistry laboratory, Faculty of Nursing, School of Health Sciences, NKUA, Athens, Greece; 3 Swetox, Karolinska Institutet, Unit of Toxicological Sciences, Södertälje, Sweden; 4 Pediatric Research laboratory, Faculty of Nursing, School of Health Sciences, NKUA, Athens, Greece; 5 IMM –Institute for Environmental Medicine, Karolinska Institutet, Stockholm, Sweden; 6 Icahn School of Medicine at Mount Sinai, New York, NY USA; 7 Karlstad University, Karlstad, Sweden; 8 Department of Clinical Science, Intervention and Technology, Karolinska Institutet, Stockholm, Sweden

**Keywords:** Natural hazards, Endocrinology

## Abstract

The increasing concern for the reproductive toxicity of abundantly used phthalates requires reliable tools for exposure risk assessment to mixtures of chemicals, based on real life human exposure and disorder-associated epidemiological evidence. We herein used a mixture of four phthalate monoesters (33% mono-butyl phthalate, 16% mono-benzyl phthalate, 21% mono-ethyl hexyl phthalate, and 30% mono-isononyl phthalate), detected in 1^st^ trimester urine of 194 pregnant women and identified as bad actors for a shorter anogenital distance (AGD) in their baby boys. Mice were treated with 0, 0.26, 2.6 and 13 mg/kg/d of the mixture, corresponding to 0x, 10x, 100x, 500x levels detected in the pregnant women. Adverse outcomes detected in the reproductive system of the offspring in pre-puberty and adulthood included reduced AGD index and gonadal weight, changes in gonadal histology and altered expression of key regulators of gonadal growth and steroidogenesis. Most aberrations were apparent in both sexes, though more pronounced in males, and exhibited a non-monotonic pattern. The phthalate mixture directly affected expression of steroidogenesis as demonstrated in a relevant *in vitro* model. The detected adversities at exposures close to the levels detected in pregnant women, raise concern on the existing safety limits for early-life human exposures and emphasizes the need for re-evaluation of the exposure risk.

## Introduction

Endocrine Disrupting Chemicals (EDCs) are anthropogenic compounds that disrupt normal endocrine action leading to adverse health outcomes. Global bio-monitoring data show that EDCs and/or their metabolites are routinely detected in the biological fluids of the entire human population including pregnant mothers^1,2^. Embryos and developing organisms are more susceptible to EDCs, due to their immature detoxification and barrier systems. In the literature, there is ample data that EDCs can interfere with the organism’s developmental programming and contribute to disorders that may manifest later in life^3,4^. Risk assessment for EDC exposures has been almost exclusively conducted on the basis of a compound-by-compound approach, although organisms are exposed to mixtures of chemicals simultaneously. In cases that the chemicals in a mixture exhibit additive or synergistic effects, the existing risk assessment approach, based on single compounds, may lead to underestimation of the health risk^3,4^. Accordingly, there is an urgent need to improve the regulatory framework by taking mixtures into account^5^. To achieve this, the EDC-MixRisk project (http://edcmixrisk.ki.se) has followed a strategy in determining mixtures of suspected or proven EDCs that are based on epidemiological evidence of associations with adversities in distinct domains of human health. For this purpose the Swedish Environmental Longitudinal Mother and child, Asthma allergy (SELMA) study has been used^6^. Next, the defined mixtures are tested in human-relevant experimental models and the resulting data are expected to aid in the development of improved tools for risk assessment.

By the use of weighted quantile sum (WQS) regression, four phthalate metabolites, namely MBP, MBzP, MEHP and MINP (among 20 suspected or proven EDCs detected in 1^st^ trimester urine/serum of more than 2,300 SELMA mothers) were found to be associated with a shorter anogenital distance (AGD) in 194 baby boys at 22 months of age^7^. A typical mixture of these four phthalates was established, called Mixture S^7^. Mixture S has then been used in an initial mouse study, at doses corresponding to 0.5x, 10x and 100x the geometric mean of levels detected in the mothers of SELMA study (SELMA levels), and it was shown to have similar effects in reducing AGD of *in utero* exposed juvenile male offspring^7^. In the present extended study, we examine the adverse outcomes from gestational exposure to Mixture S in several indices of the reproductive physiology of male and female mice offspring, at two postnatal life stages, pre-puberty and adulthood.

Phthalates or phthalate esters, depending on their molecular weight are primarily used as plasticizers, solvents and additives in many industrial and consumer products, including food containers, children toys, medical devices, pill coating and cosmetics. They consist a class of ubiquitous environmental toxicants with some of them evaluated as EDCs^3^. Inside the organism, phthalates are rapidly metabolized and most of their reproductive effects are exerted by their monoesters or their secondary metabolites, depending of the chemical structure of the parent compound^8,9^. Several epidemiological studies link early life phthalate exposure with anti-androgenic action and reproductive dysfunction, mostly in male subjects. For example, *in utero* phthalate exposure correlates with shorter AGD in young boys^10,11^, whereas no such associations were found in the few studies performed in girls^12^. On the other hand, many reports in rodents have shown that prenatal exposure to single phthalates impacts reproductive development of both males^13–16^ and females^17^.

One limitation in studies focusing on reproductive toxicity of phthalates, and EDCs at large, is that most of them have been conducted by exposing animals to single compounds and studies using mixtures are limited. In addition, in the majority of rodent studies using mixtures of phthalates, for either perinatal^13,18–23^ or longer postnatal exposures^24,25^, the components and proportion of phthalates in the mixtures did not derive from biostatistical association of relevant epidemiological data with specific adverse outcomes.

In this study, we provide improved evidence for the developmental impact of phthalates in the reproductive system, based on a mixture of four phthalate monoesters (Mixture S) derived from statistical modeling of real life human exposure and adverse outcome that was modeled in the mouse. A sex and age split-litter approach was selected in order to study siblings of both sexes at the same time. Specific objectives were to examine parameters of reproductive development, including AGD, gonadal histology and steroidogenesis-related gene expression in the mouse model. Direct effects on steroidogenesis were addressed in an *in vitro* model.

## Results

**Figure 1:**
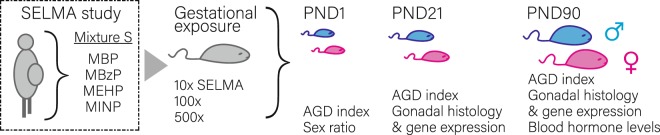
Mouse model of gestational exposure to Mixture S. Pregnant mice were exposed throughout pregnancy to vehicle or to 10x, 100x and 500x the geometric mean of SELMA study mothers’ urine/serum levels for the phthalate monoesters in Mixture S. Offspring’s anogenital index and sex ratio was monitored on postnatal day (PND) 1. A split- sex and litter design was used to study the effects of Mixture S on the reproductive system of the offspring at pre-puberty (PND21) and adulthood (PND90). Outcomes analyzed include AGD index, gonadal histology, expression of key gametogenesis and steroidogenesis regulators and circulating hormone levels.

Mixture S was determined through combined epidemiology and advanced biostatistics of the SELMA pregnancy cohort data^7^. To simulate the exposure in the SELMA cohort, in the present study pregnant mice were orally exposed daily, throughout gestation, to three different concentrations of Mixture S or the vehicle (Table 1). The effects on the reproductive system of progeny were studied on postnatal days (PND) 1, 21 and 90 (Fig. 1).

**Table 1:** Daily exposure of pregnant mice through food to Mixture S.

Chemical	Pregnant mice daily treatment (mg/kg BW)		
	10x	100x	500x
MBP	0.078	0.78	3.89
MBzP	0.041	0.41	2.05
MEHP	0.058	0.58	2.87
MINP	0.087	0.87	4.35
**Total**	0.26	2.64	13.16

x refers to the geometric mean of SELMA mothers’ levels for the chemicals in Mixture S.

### Mixture S induced changes in AGD index and gonadal weight

AGD (in humans) and AGD/cubic root of BW (“AGD index”, in animals)^26^ is an established index for monitoring sexual differentiation and lower values have been associated with early exposure to anti-androgenic or estrogenic compounds^10,11^. In the present study, significant changes in the AGD index were detected in male and female offspring *in utero* exposed to Mixture S. These changes differed by sex, dose and age (Table 2). On PND1, no significant differences were detected in males, while in females exposure to Mixture S significantly affected AGD index (Wald x^2^_females_ = 20.00, p < 0.001). Compared to the respective DMSO-treated offspring, the AGD index was significantly increased in PND1 female offspring of the 10x (0.26 mg/kg/d) SELMA level group. On PND21, the AGD index of male and female offspring was significantly modified following the prenatal exposure to Mixture S (Wald x^2^_males_ = 21.38, p < 0.001; Wald x^2^_females_ = 39.94, p < 0.001). Compared to the respective DMSO-treated offspring, AGD index was significantly decreased in pre-pubertal male and female offspring of the 10x (0.26 mg/kg/d) and 100x (2.6 mg/kg/d) SELMA level groups, but not in the 500x groups. Significant impact was also detected in adult male offspring (Wald x^2^ = 8.71, p = 0.033), where AGD index was also reduced in the 10x treated group.

**Table 2:** Reproductive indices of male and female offspring.

AGD/cubic root of BW	DMSO	10x	100x	500x
	**Males**			
PND1	1.41 ± 0.02 (34)	1.52 ± 0.04 (14)	1.42 ± 0.03 (19)	1.37 ± 0.03 (26)
PND21	3.81 ± 0.04 (30)	3.61 ± 0.07* (13)	3.56 ± 0.05^¤^ (19)	3.79 ± 0.04 (26)
PND90	6.42 ± 0.05 (21)	6.16 ± 0.08* (8)	6.43 ± 0.07 (12)	6.41 ± 0.06 (13)
	**Females**			
PND1	0.93 ± 0.02 (26)	1.06 ± 0.03^#^ (15)	0.98 ± 0.02 (14)	0.94 ± 0.02 (16)
PND21	2.29 ± 0.03 (24)	2.13 ± 0.04^¤^ (17)	2.01 ± 0.04^#^ (14)	2.27 ± 0.04 (12)
PND90	3.26 ± 0.03 (14)	3.18 ± 0.04 (9)	3.24 ± 0.04 (9)	3.24 ± 0.04 (9)
**Gonad weight/BW**	**DMSO**	**10x**	**100x**	**500x**
	**Males**			
PND21	3.05 ± 0.04 (11)	3.21 ± 0.06 (5)	2.85 ± 0.06* (7)	2.90 ± 0.05 (9)
PND90	4.41 ± 0.06 (21)	4.35 ± 0.09 (7)	4.28 ± 0.07 (12)	4.28 ± 0.07 (10)
	**Females**			
PND21	0.90 ± 0.29 (10)	0.87 ± 0.03 (8)	0.65 ± 0.04^#^ (5)	0.84 ± 0.04 (5)
PND90	1.18 ± 0.056 (14)	0.89 ± 0.07 (9)	0.78 ± 0.07^#^ (9)	1.15 ± 0.06 (9)

AGD/cubic root of BW and gonad weight/BW values for postnatal day (PND) 1, 21 and 90 male and female offspring exposed to DMSO or to 10x, 100x and 500x of SELMA mothers’ levels for the chemicals in Mixture S. In the statistics of gonad weight/bw, the phase of the estrous cycle of PND90 females was included as covariate. Values represent estimated marginal means ± SE. The number of animals is given in parentheses. *p < 0.05; ^¤^p < 0.01; ^#^p < 0.001 vs. DMSO.

Gestational exposure to Mixture S significantly modified gonadal weight in PND21 offspring of both sexes (Wald x^2^_males_ = 23.17, p < 0.001; Wald x^2^_females_ = 27.23, p < 0.001) and in adult females (Wald x^2^ = 30.89, p < 0.001). Compared to the matching DMSO-treated group, gonad weight/bw was significantly reduced in the 100x group of pre-pubertal males and females, and in the 10x and 100x groups of adult females (Table 2). No significant effect of exposure to Mixture S was detected on body weight of male offspring on any of the developmental stages examined, while on PND21, female animals of the 100x and 500x SELMA level groups had lower body weight (Wald x^2^ = 72.06, p < 0.001) (Supplement Table S1).

Exposure to Mixture S had no significant effect on the litter size or the birth sex ratio (Supplement Table S2). External genitalia and nipple retention in male pups inspected on PND10 were not affected by Mixture S exposure. The age of vaginal opening in female offspring did not differ significantly among groups (Supplement, Fig. S1). A total number of 41 adult females was sacrificed on PND90 of which 8 animals (19.5%) were at proestrous, 14 (34.1%) at estrous, 9 (22%) at methestrous and 10 (24.4%) at diestrous.

### Mixture S induced gonadal histopathology

Developmental exposures to single phthalate compounds often disturb gonadal differentiation and morphology. In the present study, *in utero* exposure to Mixture S induced morphological aberrations in the gonads of male and female offspring when examined in pre-puberty and adulthood.

**Figure 2:**
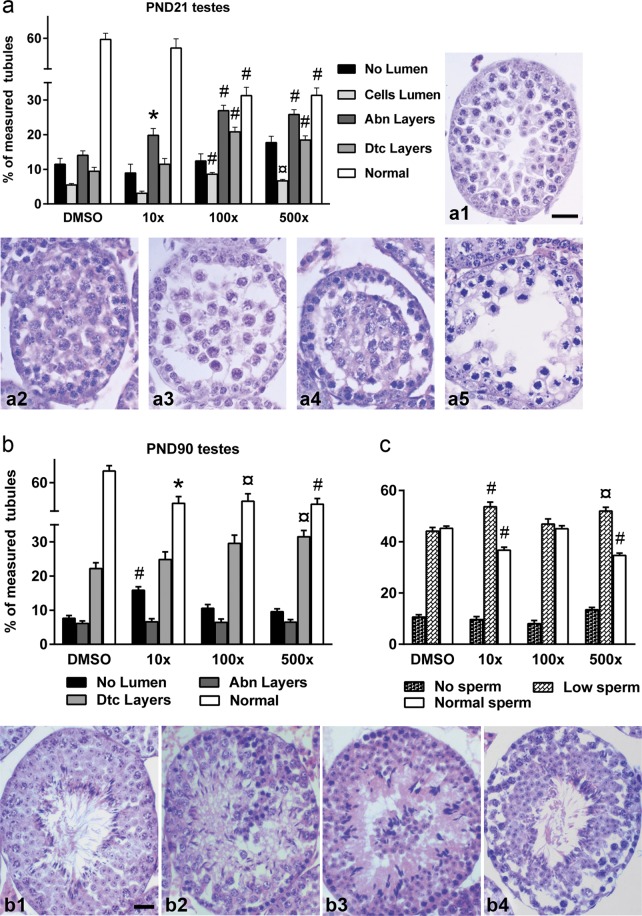
Effect of Mixture S on testicular histology of PND21 (**a**) and on testicular histology (**b**) and sperm content (**c**) of PND90 offspring exposed to 0x (DMSO), 10x, 100x and 500x SELMA mothers’ levels. Bars represent the estimated marginal means ± SE of the percent of seminiferous tubule types detected in each group. Number of animals used PND21: n = 4–10; PND90: n = 7–13. *p < 0.05; ¤p < 0.01; ^#^p < 0.001. Photomicrographs of tubules from DMSO-treated and Mixture S-treated animals at PND21 (a1–a5) and PND90 (b1–b4) showing normal tubules (a1, b1) and representative aberrations detected in experimental animals. a2, b2: no lumen; a3, b3: germ cells in the lumen; a4, b4: detached layers; a5: abnormal layers. Scale bar 20 μm.

In the PND21 male gonad, Mixture S induced aberrations in the seminiferous tubules that were more abundant in the 100x and 500x exposed groups (Fig. 2a and Supplement Fig. S2A). The detected abnormalities included tubules with thinner or disorganized layers due to missing or atypical germ cells, gonocytes inside the lumen and detached layers from the basement layer. The percentage of tubules with an abnormal layer structure was significantly increased in all groups vs. DMSO (Wald x^2^ = 53.99, p < 0.001). Furthermore, significantly increased percentage of tubules with cells in the lumen (Wald x^2^ = 60.45, p < 0.001) and detached layers (Wald x^2^ = 56.12, p < 0.001) were detected in the 100x and 500x pre-pubertal groups, with a concomitant decrease in the normally structured tubules (Wald x^2^ = 141.39, p < 0.001). Considerable histopathological changes were also witnessed in the testes of adult offspring (Fig. 2b and Supplement Fig. S2B) where all Mixture S - treated groups had significantly reduced percentage of tubules with normal histology (Wald x^2^ = 24.04, p < 0.001). The aberrations mostly detected were tubules without lumen, tubules with multinucleated germ cells or residual bodies of spermatids in the lumen, tubules with layers detached from the basement level and tubules with abnormal layers (exhibiting disorganized arrangement or missing gonocytes into the layers). Quantification and statistical evaluation of the abnormalities showed increased incidence of tubules without lumen in 10x group (Wald x^2^ = 40.02, p < 0.001) and increased tubules with detached layers in 500x group (Wald x^2^ = 15.78, p = 0.001), while these abnormalities in the 100x group did not reach significance. To have an estimation of the spermatogenic capacity of the adult testes, the tubules in the digital images were categorized as having low (less than 20% coverage), normal (at least 40% coverage) or no sperm content in the lumen. Statistical analysis showed that the percentage of tubules with low sperm content was increased in the 10x and 500x groups vs. DMSO (Wald x^2^ = 22.69, p < 0.001), while the percentage of tubules with normal sperm content was significantly decreased (Wald x^2^ = 94.51, p < 0.001) (Fig. 2c).

**Figure 3:**
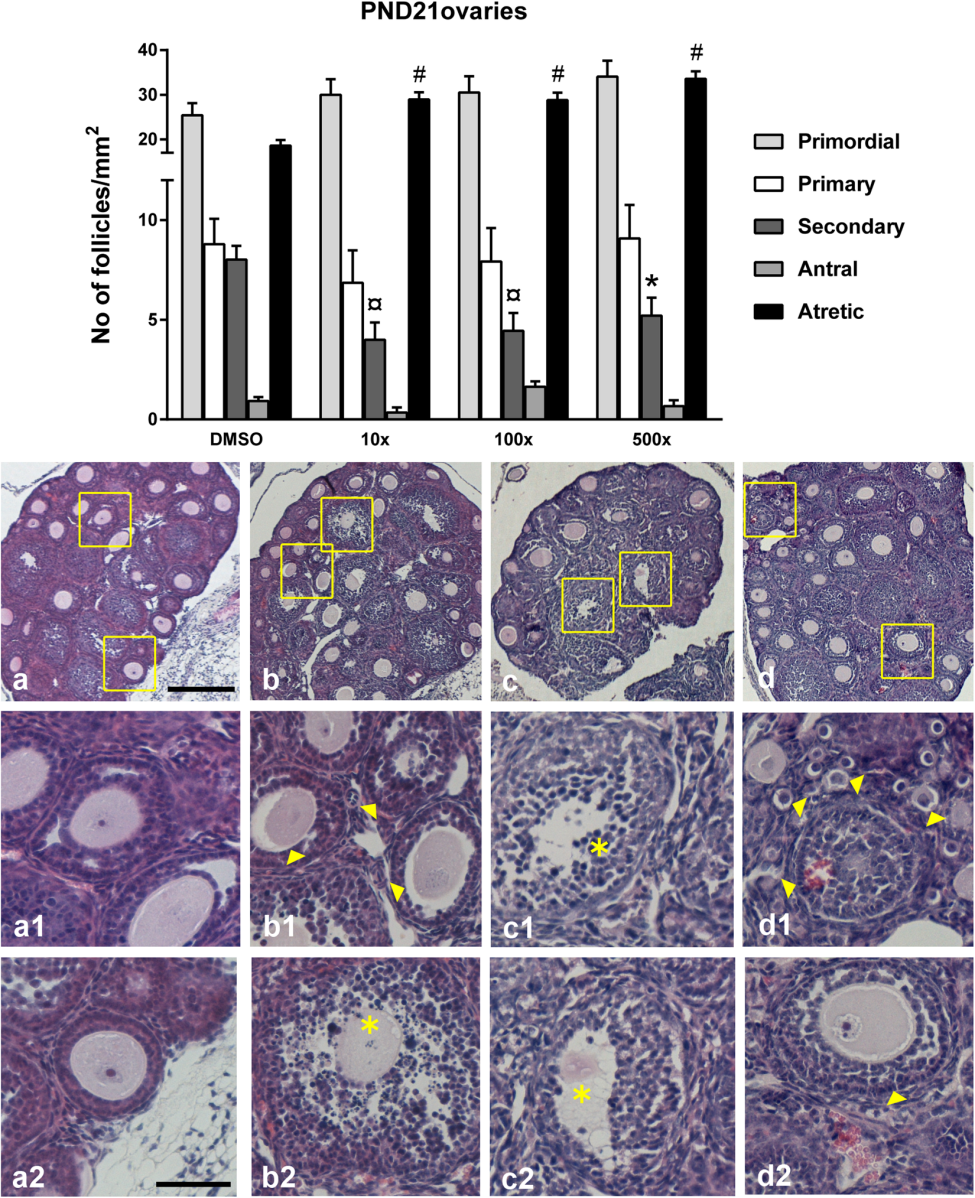
Effect of Mixture S on ovarian histology of PND21 offspring exposed to 0 x (DMSO), 10 x , 100x and 500x SELMA mothers’ levels. Bars represent the estimated marginal means ± SE of the number of follicles per mm^2^ of ovary. Significantly reduced number of preantral follicles (primary and secondary) and increased atresia were detected in all treated groups vs. DMSO. *p < 0.05; ¤p < 0.01; ^#^p < 0.001. Number of animals used: 5–9. Representative H&E stained sections of the whole ovary are provided per group ((**a**) DMSO, (**b**) 10x, (**c**) 100x, (**d**) 500x; scale bar 200 μm). Respective framed areas are shown at higher magnification below each group. Scale bar 50 μm. Yellow arrowheads denote typical atretic follicles and asterisks indicate follicles with pyknotic cells.

**Figure 4:**
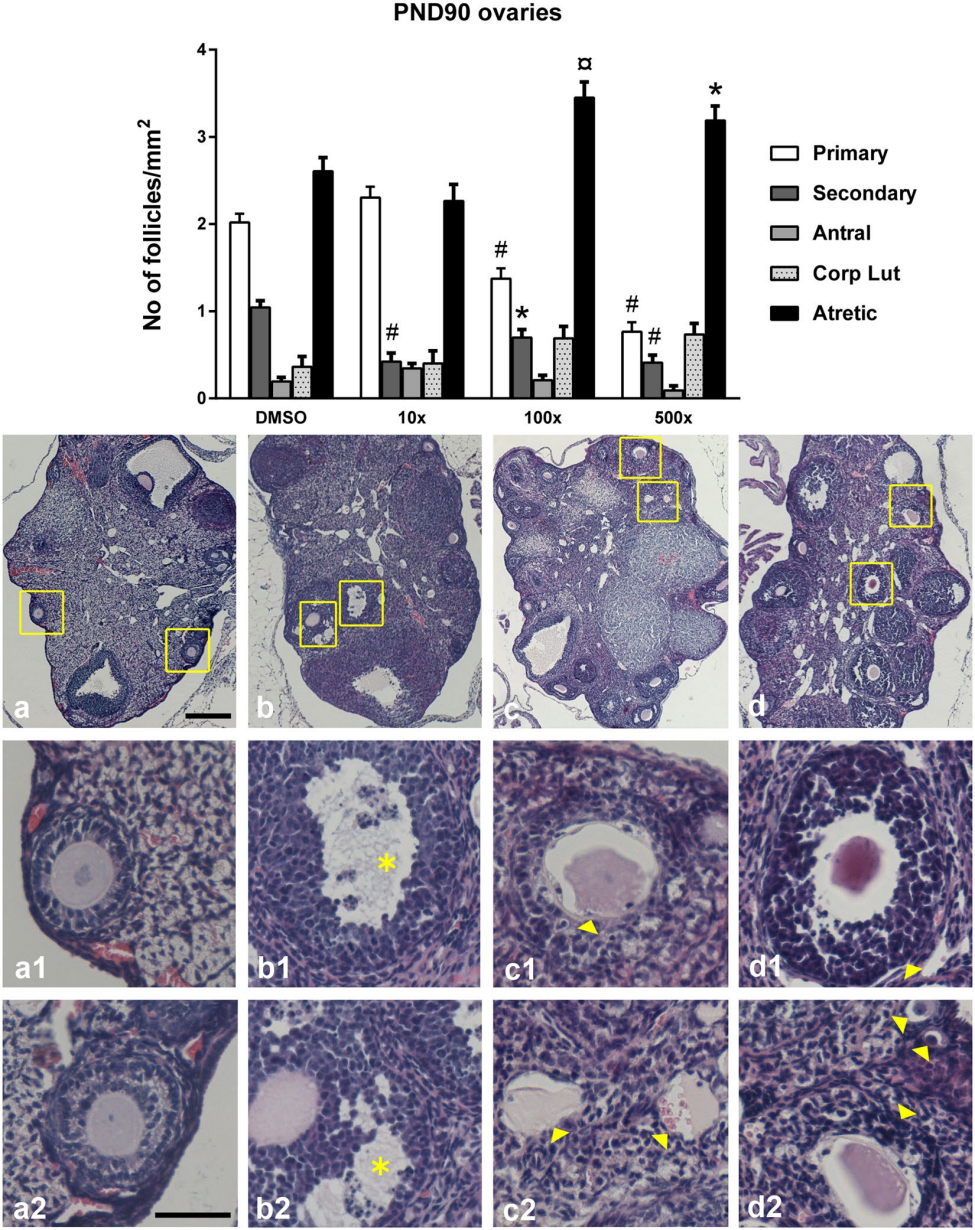
Effect of Mixture S on ovarian histology of PND90 offspring exposed to 0x (DMSO), 10x, 100x and 500x SELMA mothers’ levels. Bars represent the estimated marginal means ± SE of the number of follicles per mm^2^ of ovary. Significantly reduced number of preantral follicles (primary and secondary) and increased atresia were detected in all treated groups vs. DMSO. *p < 0.05; ^¤^p < 0.01; ^#^p < 0.001. Number of animals used: 7–12. The phase of the estrous cycle was included as covariate in the statistics. Representative H&E stained sections of the whole ovary are provided per group ((**a**) DMSO, (**b**) 10x, (**c**) 100x, (**d**) 500x; scale bar 200 μm). Respective framed areas are shown at higher magnification below each group. Scale bar 50 μm. Yellow arrowheads denote typical atretic follicles and asterisks indicate follicles with pyknotic cells.

Ovaries from PND21 offspring exposed to all doses of Mixture S (ranging from 0.26 to 13 mg/kg/d) had significantly reduced number of secondary follicles (Wald x^2^ = 17.68, p < 0.001) (Fig. 3). Importantly, they also had a significantly increased number of atretic follicles vs. DMSO (Wald x^2 = ^60.83, p < 0.001). Unhealthy follicles exhibited degenerated oocytes, atretic bodies, pyknotic nuclei or haemorrhages. The ovaries of PND90 offspring showed signs of early ovarian senescence (Fig. 4) including a reduced pool of pre-antral follicles (Wald x^2^_primary_ = 97.1, p < 0.001; Wald x^2^_secondary_ = 38.10, p < 0.001) and increased levels of atretic follicles (Wald x^2^ = 24.57, p < 0.001). Specifically, compared to DMSO, the ovaries of all Mixture S - treated groups had reduced levels of secondary follicles. The ovaries of 100x and 500x groups had also reduced primary follicles and significantly increased atresia.

### Mixture S affected gene expression involved in gonadal differentiation and steroidogenesis

**Figure 5:**
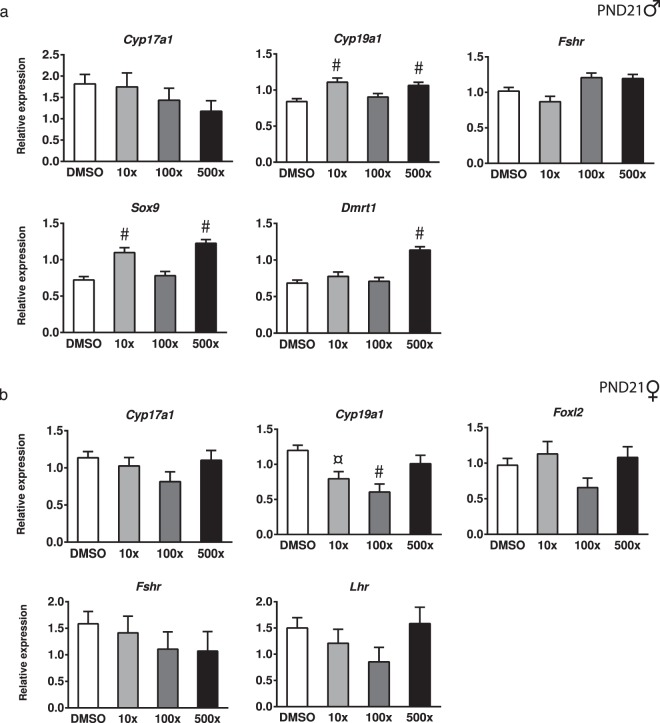
Effect of Mixture S on gonadal gene expression of PND21 male (**a**) and female (**b**) offspring exposed to 0x (DMSO), 10x, 100x and 500x SELMA mothers’ levels. Expression levels of indicated genes were evaluated by qRT-PCR and normalized to *b-actin*. Bars represent the estimated marginal means ± SE. Number of animals used: males 5–10; females 4–10. ^¤^p < 0.01; ^#^p < 0.001.

In the mouse, sex determination and gonadal differentiation are regulated by the timely expression of specific genes including *Sox9* and *Dmrt1* in males^27–29^ and *Foxl2* in females^30^. Since the role of these genes is also important during postnatal development^31–33^, their expression was examined in the premature gonads of PND21 animals exposed to Mixture S. As shown in Fig. 5a, there was a significant increase (Wald x^2^ = 66.79, p < 0.001) in the expression of *Sox9* in the testes of the10x and 500x groups, but not in the 100x group. Testicular *Dmrt1* was also increased in the 500x group of pre-pubertal offspring (Wald x^2^ = 63.70, p < 0.001). There were no significant differences among groups in *Foxl*2 mRNA in the ovaries of their female siblings (Fig. 5b).

Steroid production by the gonads is crucial for both development and function of the reproductive system. To investigate whether *in utero* exposure to Mixture S had an impact on the early expression of the steroidogenic genes prior to the maturation of the hypothalamic-pituitary-gonadal axis, we determined the expression levels of *Cyp17a1*, *Cyp19a1* and of the gonadotropin receptors *Fshr* and *Lhr* in the gonads of PND21 offspring. Expression of *Cyp19a1* in the premature testes was significantly increased in the offspring exposed to 10x and 500x SELMA levels, but not significantly in the 100x group (Wald x^2^ = 23.04, p < 0.001) (Fig. 5a). Interestingly, the same gene was significantly decreased in the developing ovaries of the 10x and 100x groups (Wald x^2^ = 22.41, p < 0.001) (Fig. 5b). No significant differences were detected in the expression of *Cyp17a1*, *Fshr* and *Lhr* in the premature gonads of either sex.

**Figure 6:**
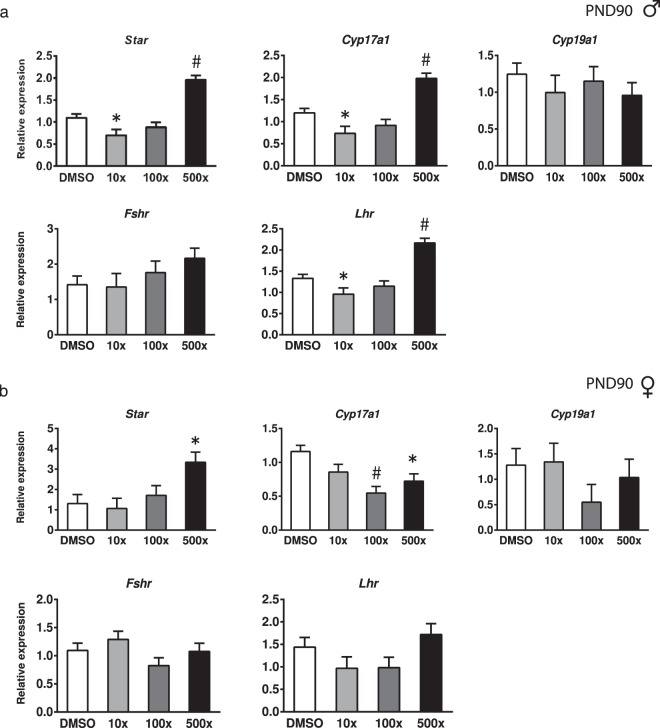
Effect of Mixture S on gonadal gene expression of PND90 male (**a**) and female (**b**) offspring exposed to 0x (DMSO), 10x, 100x and 500x SELMA mothers’ levels. Expression levels of indicated genes were evaluated by qRT-PCR and normalized to *b-actin*. Bars represent the estimated marginal means ± SE. Number of animals used: males 8–20; females 9–13. *p < 0.05; ^#^p < 0.001. In females, the phase of the estrous cycle was included as covariate in the statistics.

The long-term effects of embryonic exposure to Mixture S on the expression of key steroidogenic enzymes, and of the gonadotropin receptors were studied in the gonads of the adult offspring. In the mature testes, the mRNAs of *Star*, *Cyp17a1* and *Lhr* were significantly decreased in the 10x group and significantly increased in the 500x group, compared to DMSO (Wald x^2^ = 79.30, p < 0.001; Wald x^2^ = 54.46, p < 0.001; Wald x^2^ = 65.72, p < 0.001, respectively) (Fig. 6a). In the ovaries of PND90 offspring, *Cyp17a1* levels were significantly reduced in the 100x and 500x groups (Wald x^2^ = 23.37, p < 0.001), while *Star* mRNA was increased in the 500x group (Wald x^2^ = 12.26, p = 0.007) (Fig. 6b). No significant differences were detected in the expression of *Lhr* in the mature ovaries and of *Cyp19a1* and *Fshr* in the adult gonads of either sex.

**Figure 7:**
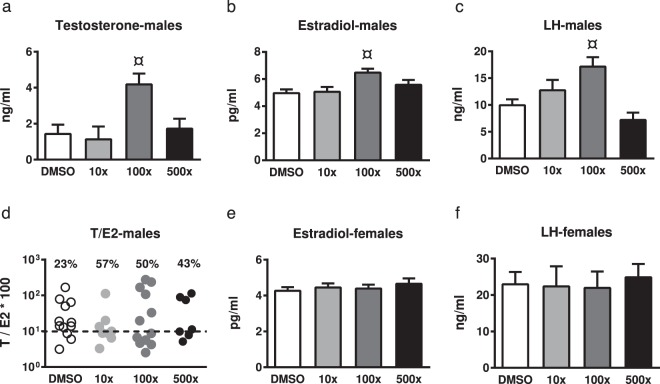
Effect of Mixture S on circulating Testosterone (T), Estradiol (E2) and Luteinizing hormone (LH) levels of PND90 male (**a–c**) and female (**e**,**f**) offspring exposed to 0x (DMSO), 10x, 100x and 500x SELMA mothers’ levels. In (**d**) the percentage of males per group exhibiting a T/E2*100 value lower than the threshold of 10 (dashed horizontal line) is indicated. Bars represent the estimated marginal means ± SE. Number of males used for T, E2 and LH: 8–16, 7–13 and 3–11, respectively. Number of females used for E2 and LH: 5–13 and 3–6, respectively. Samples of siblings were pooled for LH. In females, the phase of the estrous cycle was included as covariate in statistics. ^¤^p < 0.01.

To examine whether the observed alterations in gonadal histology and steroidogenesis - related gene expression were reflected in the circulating hormone levels of PND90 offspring, we measured plasma estradiol and LH (in males and females) and testosterone (in males). Testosterone, estradiol and LH levels were significantly increased in adult male offspring of the 100x group (Wald x^2^ = 16.07, p = 0.001; Wald x^2^ = 16.26, p = 0.001; Wald x^2^ = 21.55, p < 0.001, respectively) (Fig. 7). No significant differences were detected in the other groups of male offspring or in the female offspring. In the case of male offspring, however, approximately half of the animals in the treated groups exhibited testosterone-to-estradiol *100 ratio lower than 10. Ratios below this threshold have been associated with male infertility^34^.

**Figure 8:**
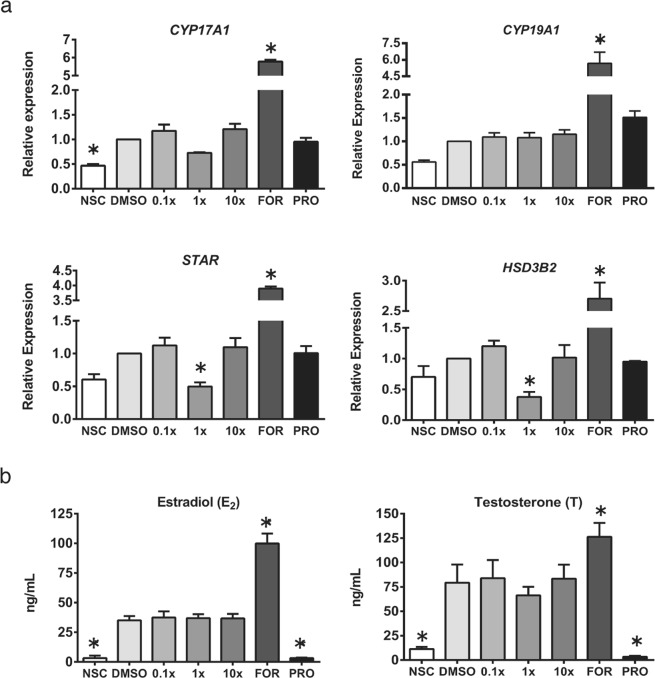
Effect of Mixture S on steroidogenesis *in vitro*. Gene expression levels of steroidogenic enzymes in H295R cells (**a**) and production of steroid hormones after 24 h Mixture S exposure (**b**). NSC – non-stimulated, non-treated control; FOR – forskolin; PRO – Prochloraz. N = 3 experiments with 2 technical replicates; data represent mean ± SEM; *p < 0.05 vs. DMSO control, One-Way ANOVA with Dunnett’s multiple comparison test.

To study whether Mixture S affected steroidogenesis directly or indirectly through adverse effects on tubuli and follicle numbers, we utilized an *in vitro* steroidogenesis assay based on the human adrenocortical carcinoma cell line H295R. H295R cells are the only human-derived cell line capable of *de novo* steroid synthesis, and importantly they are a validated guideline test system for studying chemical effects on steroidogenesis^35^. Firstly, cytotoxicity of Mixture S was examined by measurement of cell viability, and no effect was observed after 48 hours of treatment (Supplement Fig. S3). Gene expression of key steroidogenic enzymes was examined after 24 hours of exposure to Mixture S and a non-monotonic response was observed. The expression of *STAR* and *HSD3B2* was significantly decreased (p < 0.05) following 24 hours of treatment with 1x concentrations, but not with 0.1x or 10x, compared to DMSO control (Fig. 8a). The observed reduction in *CYP17A1* expression level did not reach statistical significance (p = 0.132). No change in response to Mixture S was observed for *CYP19A1*. As expected, treatment with the steroidogenesis stimulator Forskolin (FOR) was able to increase gene expression compared to DMSO for all genes while the inhibitor of steroidogenic enzyme activity Prochloraz (PRO) was not different from DMSO control (Fig. 8a).

Levels of estradiol and testosterone were measured in conditioned media after 24 hours of Mixture S exposure (Fig. 8b). No significant effect on hormone levels was seen for any concentration of the Mixture S. FOR treatment stimulated hormone production as expected, while PRO reduced steroid levels. Similarly, examination of androstenedione, progesterone, 17α-hydroxyprogesterone and dehydroepiandrosterone levels showed no effect after Mixture S exposure (Supplement Fig. S4).

## Discussion

Most previous studies conducted in animals have reported reproductive aberrations caused by single phthalates at doses higher than the human exposure rate. The present study examined the *in utero* reproductive toxicity upon exposure to a mixture of phthalates, instead of single compounds, which better mimics the real life conditions required for appropriate risk assessment^5^. The use of a mixture of phthalate monoesters instead of diesters was considered to be representative of the human exposure in pregnant women of the SELMA cohort, whose monoesters were used to determine the Mixture S^7^. Mixture S components and proportions were defined from an integrated approach that has implemented data of EDC concentrations of 20 compounds in pregnant women of the SELMA study with an index of early sexual differentiation (AGD) in their male offspring. In the frame of the EDC-MixRisk project, the impact of Mixture S on the mammalian reproductive system is extensively studied for the first time by using an *in vivo* and an *in vitro* model. Our results in the mouse model show that Mixture S, at doses 0.26, 2.6 and 13 mg/kg/d (10, 100 and 500 times the estimated geometric mean of SELMA mothers’ exposure), impacted on reproductive development of the exposed offspring and induced gonadal dysfunction that appeared at later life stages. Effects included reduction in the AGD index in young offspring, reduced gonadal weight, increased aberrations in gonadal histology and altered expression of genes regulating gonadal growth and steroidogenesis. The aforementioned effects, detected in pre-puberty and/or adulthood, were apparent in both sexes, though more pronounced in males, and exhibited a non-monotonic pattern, characteristic to many EDCs including phthalates^36^. The results of the *in vitro* model support direct effects of Mixture S on steroidogenesis with altered gene expression of key enzymes that initiate the steroidogenic synthesis pathway.

AGD, twice in length in males compared to females, is an index of normal male sexual differentiation and of reproductive toxicity upon gestational exposure to phthalates and other EDCs^37,38^. Epidemiological studies, including the SELMA study cohort^10,11,39^, report reduced AGD in young boys gestationaly exposed to phthalates. In the present study, AGD index was significantly reduced in the PND21 male offspring exposed to the lower doses of Mixture S (0.26 and 2.6 mg/kg/d) but not to the higher dose of 13 mg/kg/d, as well as in the PND90 males exposed to the lower dose of 0.26 mg/kg. This effect appears non-linear and is compatible with the proposed non-monotonous action of various EDCs^36^ and with the reported anti-androgenic properties of the Mixture S components^40^. Previous studies in male mice perinatally exposed to single phthalates also reported shorter AGDs^14–16^ though the effect was detected at much higher exposures than in the present study^14,16^ and at different ages (PND60 and PND100). Of note, AGD is not at present equally well established marker in mice as it is in rats, where AGD reduction associates with phthalate-induced inhibition of fetal testosterone production^41^. In male rats^42,43^, but not mice^44–47^ phthalates inhibit testosterone production by the fetal testis. So, a direct association between fetal testosterone levels and AGD length cannot be made. However, despite the existing species differences, phthalate-exposed mice display some typical testicular changes of the rat (impaired seminiferous cord formation and gonocyte multinucleation)^45^, as well as alterations in the expression of testis genes involved in lipid metabolism and cholesterol synthesis^41^.

Previous studies in female rodents have shown that *in utero* exposure to lower-than-normal levels of androgens reduces AGD of the offspring^48–50^. However, the evidence for phthalate-induced AGD changes in females exposed *in utero* is varying^17^. In agreement with our findings for PND21 offspring, reduced AGD was also detected upon gestational exposure of mice to a mixture of phthalates and phenols^22^ that contained some of the phthalate monoesters of Mixture S. On the other hand, a study in female rats prenatally exposed to an anti-androgenic mixture containing phthalates reported no effects on AGD^51^. An explanation for the increased AGD index detected in the group of 10x females at PND1 could relay on the small -but important- differences among PND1 pups in the exact gestational length and the hours after birth at the time of AGD measurement.

SOX9 and DMRT1 are key regulators of sex determination and maintenance of the gonadal sex in males^27,28,32^. In the fetal testis, SOX9 specifies Sertoli cells to shape the seminiferous cords and set the basis for spermatogenesis. *Sox9* and *Dmrt1* expression decreases significantly around the 3rd postnatal week in the murine testis^29,31^ and this signifies the cessation of Sertoli cells’ proliferation and the onset of their differentiation, that in turn is fundamental for the maturation of germ cells and the final gonadal size^52^. In the present study, we detected increased levels of *Sox9* and *Dmrt1* in the PND21 offspring of the 10x and 500x groups that are suggestive of a delayed gonadal maturation. In line with our findings, Jobling *et al*.^53^ reported a delay in the early (PND6) postnatal re-expression of Dmrt1 in germ cells of *in utero* exposed to DBP rats. Previous studies have shown that gestational exposure to DEHP reduces *Sox9* in the testes of fetuses and young mouse offspring^54,55^. Differences in the properties of monoesters vs. diesters^40^ and the differential effects of chemicals within a mixture could explain this discrepancy. Nevertheless, it appears that the timely impaired *Sox9* expression in the developing testis affects testicular maturation and the histological observations of both studies comply with this.

*In utero* exposure to Mixture S had a sexually dimorphic and non-monotonic effect on the expression of *Cyp19A1* (aromatase) in developing gonads. *Cyp19* levels were increased in the testes while decreased in the ovaries. Aromatase activity during development is mainly located in Sertoli cells, rendering them the major source of E2 in the immature male gonad. During testicular development, E2 signaling via ER-alpha in Sertoli cells is required for normal maturation of these cells, which in turn will maintain the adult spermatogenesis^56^. Based on the above, the detected *Cyp19* increase in the immature testes of 10x and 500x offspring implies improperly increased E2 levels that could have impacted Sertoli cells’ maturation and, prospectively, adult gametogenesis.

In the rodent ovary, aromatase expression and activity, mainly localized in primary and secondary follicles, is normally increased between the 1st and 3rd week of life^57^. This FSH-driven increase is considered important to ensure the E2 required for the programming of the adult ovarian function^58^. The reduced *Cyp19* levels we see in the PND21 ovaries of the 10x and 100x offspring could result in reduced E2 availability during the time of postnatal ovary development. Given that both E2 and T stimulate the FSH-driven aromatase increase^59^ and since we did not detect any change in *Fshr* levels, we hypothesize that the detected reduction of *Cyp19* in the pre-pubertal ovary rather reflects a local disturbance in the levels of sex hormones.

The testicular aberrations, detected in the pre-pubertal offspring exposed to Mixture S and retained in adults, support the programming effects of early life toxicants in reproductive physiology. The observed histopathology is in line with previous studies, perinatally exposing mice to single phthalates^14–16^, whose monoesters also exist in Mixture S, and with a recent study, exposing mice from gestation until adulthood, to a mixture of phthalates and alkylphenols^24^. Although direct comparisons cannot be made due to the different administration protocols, the different mixtures components and the differential effects of single compounds vs. their mixtures^60^, it is worth noting that in our study the impairments were detected at substantially lower concentrations of phthalates, compared to the previous gestational exposures to single chemicals. Specifically, the impairing doses of MEHP in Mixture S ranged from 58 μg/kg/d to 2.93 mg/kg/d, compared to 300 and 750 mg/kg/d of DEHP^15,16^ in the previous studies. Similarly, MBP impairing exposure in Mixture S ranged from 78 μg/kg/d to 3.9 mg/kg/d, compared to 100 mg/kg/d of DBP^14^. This supports the view that mixtures of EDCs can have cumulative effects on the reproductive development. Overall, the later-life impact of Mixture S on testicular histology was more pronounced in the 10x (0.26 mg/kg/d) and 500x (13 mg/kg/d) groups, implying again that the action of the phthalates did not follow a linear dose-response pattern.

Gestational exposure to Mixture S affected adult steroidogenesis of the male gonad also in a non-monotonic manner. The LH-regulated expression of *Star* and *Cyp17*, was impaired, resulting in either *Star*, *Cyp17* and *Lhr* down-regulation or up-regulation in the 10x and 500x offspring, respectively. These findings suggest that the regulatory feedback loop in Leydig cells^61^ is not properly developed in these offspring. The detected alterations in adult offspring steroidogenesis could reflect the disordered expression of *Sox9*, *Dmrt1* and *Cyp19* in the testis of their PND21 siblings. As previously mentioned, the timely and proper expression of these genes is required for the programming of adult steroidogenesis. In addition, the reduced sperm content seen in the adult 10x and 500x offspring is compatible with their overall gonadal dysfunction that implies reduced spermatogenesis, although detailed investigation of sperm quality was not performed in this study. The increased *Lhr* expression in the testes of 500x offspring is not expected to coincide with analogous increases in serum LH or testosterone levels, according to the hormonal phenotype of adult mice over expressing LHR (YHR^+^ mice)^62^. Although circulating testosterone and estradiol levels in adult 10x and 500x offspring did not differ significantly, compared to non-exposed controls, there was a decrease in the serum T/E2 ratio that in humans has been associated with male infertility^34^. The most striking alteration in circulating hormone levels, however, was detected in the adult 100x group in which plasma levels of T, E2 and LH were significantly elevated. Interestingly, this group exhibited limited changes in adult testes histology and no changes in the steroidogenic genes investigated. This seemingly paradox could be explained based on previous observations that the impact of phthalates in testicular steroidogenesis and gametogenesis  are not necessarily interrelated^63^.

The E2 to T imbalance due to reduced aromatase could have contributed to the histopathology detected in PND21 ovaries. In view of the fact that the total number of follicles is determined at the pre-pubertal period, the significant elimination of pre-antral follicles, in combination with the increased atresia, could have led to the reduced ovarian weight detected in the adult offspring. The overall ovarian histopathology in the adult offspring is reminiscent of the precocious ovarian insufficiency described in humans^64^. The detected abnormalities at exposures ranging from 0.26 to 13 mg/kg/d of the Mixture S, are in accordance with the defects reported upon gestational exposure of rodents to single phthalates (mostly diesters) though at doses from 2 mg and above^65,66^ (for a review see^17^) or to mixtures including phthalates^21^.

Similar to the males of this study, *in utero* exposure to Mixture S affected adult ovarian steroidogenesis by targeting the expression of *Cyp17*, whilst to the opposite direction. The decreased expression of this testosterone producing enzyme could consequently reduce ovarian testosterone levels, known to support the integrity of primary and secondary follicles against atresia^67^. Accordingly, pre-antral follicles were significantly reduced and the atretic follicles were increased in the 100x and 500x groups. The increase of the upstream mediator of steroidogenesis *Star* in the group of 500x females could represent a compensating (homeostatic) response to the decreased *Cyp17* expression.

Many of the findings from our mouse studies regarding steroidogenesis could result from the observed reduced numbers of follicles or tubuli capable of steroidogenesis in the exposed animals. However, our *in vitro* results suggest that Mixture S has a direct effect on steroidogenesis. In the human derived adrenal cell line capable of *de novo* steroidogenesis, we observed significant inhibitory effects of Mixture S on the expression of *HSD3B2* and *STAR*, and a small reduction in *CYP17A1*. As in mouse studies, the effect did not follow a traditional dose-response. Furthermore, the changes in gene expression did not lead to major alterations in steroid hormone secretion, in agreement with the mouse studies. In the cell assay, this may be explained by an inadequate period of time from exposure to measurement, where changes in hormone production may take longer to occur as a result of alterations in enzymatic gene expression compared to direct inhibition of the enzyme as observed with PRO treatment^68^. Future studies should test long-term effects of mixture S exposure on steroid secretion *in vitro*. Collectively, these findings suggest that the effects of Mixture S on steroidogenesis are based on inhibition of gene expression and can be captured both in human *in vitro* models and mouse *in vivo* systems suggesting a general mode of action.

Further, the results corroborate the validity of the mouse model as a tool to provide causative evidence for associations observed in epidemiological studies. In the frame of EDC-MixRisk Project, a whole mixture approach linking data from population epidemiology and experimental animals is applied for evaluating the risk exposure to chemical mixtures, based on sufficient similarity of the exposures. To this direction, the data from the mouse offspring -exposed to similar concentrations of Mixture S components as detected in pregnant women- could be used to recalculate the rate of pregnant women at risk to have boys with aberrant reproductive growth. A paradigm is provided in Bornehag *et al*.^7^. Although extrapolation of experimental data to humans should be done with caution, the detected adversities induced by Mixture S, at doses ranging from below the minimal risk to occupational exposure, raise concern on the existing safe limits for early-life human exposure and denotes the need for re-evaluation of the exposure risk.

## Methods

### Mixture composition and exposure rates

The process to define Mixture S components and composition is described in Bornehag *et al*.^7^. In brief, four phthalates (DBP, DBzP, DEHP, DiNP) among 20 (proven or suspected) EDCs identified in 1^st^ trimester urine/serum of pregnant women in the SELMA study were found as bad actors for a shorter AGD in 194 baby boys at 22 months of age, by the use of weighted quantile sum (WQS) regression^69^. The daily intake for the bad actors was calculated using urinary geometric mean concentrations in 2,313 SELMA mothers^70^. The serum levels of the phthalates in SELMA mothers were estimated from the daily intake of the diesters and used to construct mixing proportions of their active monoesters^40^. Based on the above process, Mixture S is composed of 33% MBP (2.3 E-08 mol/L), 16% MBzP (1.1 E-08 mol/L), 21% MEHP (1.5 E-08 mol/L) and 30% MINP (2.1 E-08 mol/L). In the present study, pregnant mice were exposed throughout pregnancy to 0, 0.26, 2.6 and 13 mg/kg/d of the Mixture S (representing 0x, 10x, 100x, 500x, of the geometric mean of pregnant women’s serum levels for the same chemicals). The daily exposure of pregnant mice to each monoester of the Mixture S, per dose, is shown in Table 1.

The monoesters used in Mixture S were purchased from the following sources: MBP (95% purity) and MEHP (90% purity) were purchased from TCI, Tokyo Chemical Industry Co., Ltd (Japan). MBzP (98% purity) was purchased from Sigma-Aldrich Inc (St. Louis, MO, USA). MiNP (96% purity) was obtained from Toronto Research Chemicals (North York, ON, Canada). 1 M stock solution of Mixture S was prepared in DMSO (99.9% purity, Sigma-Aldrich Inc.) using the monoesters at the defined proportions. Stock was aliquoted in volumes of 10 μl in 0.2 ml 100% polypropylene Eppendorf tubes and stored at −20 °C until use. For the preparation of 10x, 100x and 500x working solutions adequate dilutions were made in PBS.

### Animals

All animal handling and experiments were conducted in accordance to the European Communities Council Directive of 22 September 2010 (2010/63/EU) and the experimental protocol was approved by the Ethical Committee of the Prefecture of Attica-Veterinary department (#4783). All efforts were made to minimize animal suffering and to reduce the number of animals used.

Two month old C57/BL6 mice, purchased from the Hellenic Pasteur Institute (Athens, Greece) were allowed to acclimatize for 2 weeks before they were used as breeders. Animals were maintained in polypropylen cages under a 12-hour light/ dark cycle, at 22 ± 2 °C, with 55 ± 10% humidity. Phytoestrogen-deficient pellet food (Altromin 1324 P, Lage, Germany) and tap water were available *ad libitum*. Females at proestrus/estrous were mated overnight with experienced males (2:1). In the next morning (potential gestational day 0.5), the females with plug were randomly distributed to four groups and were fed daily from gestational day 0.5 to delivery as follows: the vehicle-control group (DMSO in PBS; DMSO intake did not exceed 0.25 μl/gr bw/d), the 10x of the SELMA study mothers’ exposure group (0.26 mg/kg/d of Mixture S), the 100x group (2.6 mg/kg/d of Mixture S) and the 500x group (13 mg/kg/d of Mixture S) (see also Table 1). The Mixture was administered to the individually- housed dams via organic cornflakes that the animals consume willingly and readily. Each animal was offered daily one flake onto which the exact volume of the diluted mixture dose was previously pipetted and left to dry. The experimenter inspected the immediate and complete consumption of the cornflake by each animal. The lack of phthalate contaminants in the mice food or water was tested in a pilot study prior to the experiments. Mice dams that were maintained in Altromin 1324 P diet were fed with Mixture S or the vehicle (provided onto the organic corn flakes) and sacrificed one hour later. Trunk blood was collected one hour later and analyzed for the phthalate monoesters of Mixture S^7^. No phthalate monoesters were detected in the animals maintained in Altromin diet and consumed the flake with the vehicle (data not shown).

The body weights of dams were monitored and recorded every 3 days to verify pregnancy, inspect incidents of miscarriage and adjust Mixture intake. At PND1, pups’ number, sex, BW and AGD were recorded. Offspring were weaned at PND21, ear marked and separated by sex. A split-litter design was followed so as a number of animals from each litter was analyzed on PND21 and their siblings on PND90. The number of litters used per treatment was: DMSO = 9, 10x = 6, 100x = 5 and 500x = 6. For sacrifice, animals were deeply anesthetized with isoflurane and then decapitated; trunk blood was collected for hormone level determination.

### Measures of reproductive physiology

On PND1, the offspring genitourinary area was photographed in order to precisely measure AGD (the length from the center of the genital papilla to the center of the anus) using Image J (v 1.49). Each newborn pup was placed by its back on an inclined surface (upper body downwards), so that the external genitalia could be better captured in the photograph. A ruler was placed parallel to the pup body. A digital camera was used at a fixed height using a photograph mount (10 cm from table surface), lighting conditions and aperture settings were kept the same for all photographs. Pups were always manipulated after removal of the mother. On PND 21 and 90, AGD was measured using a digital caliper by the same observer, who was unaware of the animals’ treatment. The ratio of AGD measurements by the cubic root of the respective BW was used at all ages to calculate the AGD index^26^. On PND10, male pups were observed for nipple retention. Female offspring were checked daily for vaginal opening (VO) starting from PND24. Body weight was monitored from PND21 onwards at regular intervals until PND90. The phase of estrus cycle of PND90 female offspring was evaluated at sacrifice by vaginal cytology. The vaginal smears were examined under light microscopy and classified to proestrous, estrous, metestrous or diestrous. Following euthanasia the left gonad from each animal was weighted before storage at −80 °C.

### Analysis of gonadal histology

The right testis of each animal was fixed in Bouin’s solution for 3 (for PND21) or 6 (for PND90) hours and blocked in paraffin. Two central 4μm-thick cross-sections, spaced 40 µm from each other, were stained with hematoxylin and eosin (H&E). At least one testis from each litter per group and age was included in histomorphometry. The sections were digitally photographed and 4–5 photo frames (using the 10x objective lens) per section were taken for the analysis. Only round shaped seminiferous tubules that were completely visible in each photo frame were considered. The tubules were classified as normal or abnormal exhibiting one or more of the following characteristics: tubules without lumen, tubules with abnormal layering (having thinner or scrambled layer arrangement due to missing or atypical, gonocytes), tubules with detached central layers from the basement layer, or tubules with germ cells (exfoliating or multinucleated) or residual bodies of spermatids in the lumen. Additionally, in digital images obtained from all quadrants of the PND90 testes, the sperm content of the lumens was categorized as normal (at least 40% coverage), low (less than 20%) or absent (no sperm in the lumen). The number of tubules in each category was expressed as % of the total tubules measured per section. The average of the two sections from each animal was used in the statistical analysis. In all cases, at least 80 tubules were evaluated per gonad. All measurements were performed by the same observer, blind to the treatment groups.

The right ovary of each animal was fixed in 4% paraformaldehyde overnight at 4 °C, dehydrated and embedded in paraffin. Ovaries were serially cut at 4 µm and stained with H&E. At least one ovary from each litter per treatment and age was used in histomorphometry. The relative abundance and types of follicles in the ovaries were evaluated on every 10^th^ (for PND21) or 15^th^ (for PND90) section under a bright-field microscope. Follicles were annotated as primordial (oocyte partially or completely surrounded by flattened pre-granulosa cells), primary (oocyte surrounded by a single layer of cuboidal granulosa cells, GC), secondary (oocyte surrounded by two or more layers of granulosa cells, without an antrum), antral follicles, or corpus luteum. To avoid double-counting, only healthy follicles with a clearly visible oocyte nucleus were counted. Follicles were considered atretic if they presented two or more of the following characteristics: degenerated oocyte, GC layers or follicular antrum containing at least five pyknotic nuclei or atretic bodies, GC layers detached from the basement membrane, or broken basement membrane. The total number of follicles in each section was divided by the section area (mm2) to account for any differences in ovarian size between animals. The ratio of follicle number per area for each type of follicle in each ovary was used in statistical analysis. All sections were independently evaluated by two investigators unaware of the treatment group (inter investigators’ bias was <10%).

### Hormonal determination

Circulating hormone levels were quantified in collected plasma using ELISA assay kits according to the manufacturers’ instructions. Mouse-specific luteinizing hormone (LH) kit (E-EL-M0057) and testosterone (T) kit (E-EL-0072) were purchased from Elabscience. The intra- and inter-assay coefficient of variation [CV] was <10% for LH and <15% for T and the lower limit of detection [LOD] was 0.28 and 0.1 ng/ml, respectively. Estradiol kit (EA1008590) with CV of 10.4% and LOD ≤ 3 pg/ml was from OriGene Technologies Inc.

### Gene expression analyses

Total RNA was extracted from frozen gonads using Trizol reagent (Life technologies) according to the manufacturer’s protocol. Yield and purity of isolated RNA was determined spectrophotometrically. All samples had A260/A280 ratio between 1.80 and 2.10. RNA integrity was assessed by electrophoresis on agarose gels. First strand cDNA for the PCR amplification was prepared from 0.5 µg total RNA using PrimeScript™ RT Reagent Kit - Perfect Real Time (Takara). cDNA was diluted 1:40 and 5 μl were used for each quantitative polymerase chain reaction (qPCR) performed on 96-well plates, using the Mx3005P Real-Time PCR System (Agilent) and the SYBR Select Master Mix (Applied Biosystems). Primers were selected from the PrimerBank database unless otherwise noted (Supplement Table S3**)**. Reactions were prepared in duplicate and were subjected to an initial denaturation at 95 °C for 3 minutes, followed by 40 cycles of 95 °C for 3 seconds, 59 °C for 30 seconds and 72 °C for 45 seconds. Product specificity was assessed by melting curve analysis and selected samples were run on 2% agarose gels for size assessment. Data were collected as raw CT values and fold changes in gene expression were calculated using the 2−ΔΔCT method^71^ with *β-actin* as the internal reference gene.

### *In vitro* steroidogenesis assay

Human adrenocortical carcinoma cell line H295R (ATCC^®^ CRL-2128^TM^, VA, USA) was cultured in Dulbecco’s Modified Eagle’s Medium with Ham’s nutrient mixture F12 (DMEM/Ham-F12; Gibco^®^ by Life Technologies^TM^, NY, USA) supplemented with 2% penicillin-streptomycin (Gibco^®^ by Life Technologies^TM^, NY, USA), 2.5% Nu-serum (Corning Incorporated, MA, USA) and 1% Corning^®^ ITS^TM^ + Premix Universal Culture Supplement (Corning Incorporated, MA, USA) in 5% CO_2_ at 37 °C. Mixture S stock solution (1 µM in DMSO) was diluted to 1000-fold experimental stocks that were further diluted to cell culture medium for the exposures. Volume of DMSO was kept constant, 0.1%, in all groups. Effect of mixture exposure on H295R viability was measured using CellTiter-Glo® Luminescent Cell Viability Assay from Promega (WI, USA) following manufacturer’s instructions. In brief, the H295R cells were seeded in 96-well plates in triplicates, incubated overnight, and treated with 0.1x, 1x, 10x, 100x and 1000x concentrations of Mixture S. Solvent only (0.1% DMSO) and “no treatment” were included as controls. After 48 h of exposure, CellTiter-Glo® Reagent was added and luminescence recorded using Tecan Infinite M200 Pro^TM^ (Männedorf, Switzerland). Three independent experiments were performed and data is presented as percent viability of solvent control. Steroidogenesis assay was carried out following a modified protocol developed by Karmaus *et al*.^72^. Cells were seeded into 12-well plates and the following day steroidogenesis was stimulated with 10 µM forskolin (FOR) for 48 h. Subsequently, media were changed to treatments: Mixture S at concentrations 0.1x, 1x and 10x, and controls Forskolin (10 µM), Prochloraz (3 µM), vehicle (0.1% DMSO) and non-stimulated, non-treated control (NSC). Treatments were performed in duplicate in one assay, and the assay was repeated thrice. After 24 h exposure, conditioned media was collected for steroid hormone analysis and the cells were collected for gene expression analysis. Steroids were measured by ultra-performance liquid chromatography tandem mass spectrometry (UPLC-MS/MS) as described before^73^. To determine the concentration of androstenedione, media samples were diluted 1:100 in PBS while remaining steroids were quantified in non-diluted media. Isotopically labeled analogues (^2^H or ^13^C) were used as internal standards, and internal quality controls (n = 3) and calibrators (n = 9) with known steroid content were also prepared. Samples were analyzed by liquid chromatography using an Acquity^TM^ (Waters, MA, USA) coupled to a Xevo^TM^ TQ-S mass spectrometer (Waters, MA, USA). Data processing and steroid quantification was performed in the software TargetLynx^TM^ (Waters, MA, USA) as described^73^.

For analysis of steroidogenesis enzyme expression, total RNA was isolated using RNeasy Mini Kit (Qiagen, Hilden, Germany) and 500 ng was reverse transcribed to cDNA using iScript^TM^ cDNA synthesis Kit from Bio-Rad (CA, USA). qPCR analysis was executed with SYBR^®^ Green Supermix and the CFX96^TM^ Real-time System from Bio-Rad (CA, USA). Primers were designed using NCBI primer blast and purchased from Sigma (Supplement Table S2). Data were processed with Livak’s method^71^ and normalized to the housekeeping gene *HPRT1*.

### Statistics

For the mouse studies, the data were analyzed by Factorial analysis through Generalized Linear Models using SPSS 22.0. Treatment was set as the independent variable, litter was nested into the treatment and the estrous cycle was included (when applicable) as covariate. The sequential Bonferroni post-hoc test was used (when appropriate) for pairwise comparisons. The results in this model are provided as estimated marginal means ± standard error (SE). Significance was accepted for p values < 0.05. Steroidogenesis assay data were analyzed with one-way ANOVA and in case of significant findings, treatments were compared to DMSO-control using the Dunnet’s post hoc test using GraphPad Prism 6 (GraphPad Software Inc, CA, USA).

## Supplementary information

Repouskou et al_Supplement

## Data Availability

The datasets generated and/or analyzed during the current study are available from the corresponding author upon reasonable request. All data generated or analyzed during this study are included in this published article.
