# Supplementary material for: Gestational exposure to an epidemiologically defined mixture of phthalates leads to gonadal dysfunction in mouse offspring of both sexes
Source: Sci Rep. 2019 Apr 23;9:6424. doi: 10.1038/s41598-019-42377-6 (PMC6478857; doi:10.1038/s41598-019-42377-6)
Supplement: Supplementary file 1 — Repouskou et al_Supplement [file 41598_2019_42377_MOESM1_ESM.pdf]

Title: **Gestational exposure to an epidemiologically defined mixture of phthalates leads to gonadal dysfunction in mouse offspring of both sexes**

Anastasia Repouskou, Emily Panagiotidou, Lydia Panagopoulou, Pernilla Larsdotter Bisting, Astrud R. Tuck, Marcus O.D. Sjödin, Johan Lindberg, Evangelos Bozas, Joëlle Rüegg, Chris Gennings, Carl-Gustaf Bornehag, Pauliina Damdimopoulou, Antonios Stamatakis, Efthymia Kitraki.

SUPPLEMENTAL TABLES & FIGURES

Table S1. Body weights of male and female offspring on PND1, PDN21 and PND90

**Body weight** (grams)	**Males**			
	**DMSO**	**10x**	**100x**	**500x**
PND1	1.57±0.02 (34)	1.64±0.03 (14)	1.51±0.02 (19)	1.50±0.02 (26)
PND21	7.76±0.10 (30)	8.01±0.15 (13)	7.49±0.11 (19)	7.48±0.09 (26)
PND90	23.53±0.20 (21)	24.17±0.32 (8)	23.28±0.27 (12)	24.21±0.24 (13)
	**Females**			
PND1	1.52±0.03 (26)	1.59±0.04 (15)	1.47±0.03 (14)	1.42±0.03 (16)
PND21	8.14±0.09 (24)	8.21±0.11 (17)	7.31±0.11# (14)	7.23±0.11# (12)
PND90	18.52±0.22 (14)	18.01±0.27 (9)	17.82±0.25 (9)	18.8±0.25 (9)

Values represent estimated marginal means ± SE. The number of animals is given in parentheses.
# p< 0.001 vs. DMSO.

Table S2. Number of litters used, litter size and % of male offspring per group.

	**DMSO**	**10x**	**100x**	**500x**
Number of litters	9	6	5	6
Litter size (mean ± SEM)	6.78 ± 0.66	5.33 ± 0.92	6.6 ± 0.51	7 ± 0.45
Male offspring (% of total offspring)	55.7	43.8	57.6	61.9

Table S3. qRT-PCR primers used in the study

Gene	***Forward primer (5’-3’)***	***Reverse primer (5’-3’)***	*PrimerBank ID / citation*
β-actin	GGCTGTATTCCCCTCCATCG	CCAGTTGGTAACAATGCCATGT	6671509a1
Star	GTCATCAGAGCTGAACACGG	GGTGGTTGGCGAACTCTATC	designed using NCBI software
Cyp17a1	CTCCAGCCTGACAGACATTCTG	TCTCCCACCGTGACAAGGAT	6681099a1
Cyp19a1	ATGTTCTTGGAAATGCTGAACCC	AGGACCTGGTATTGAAGACGAG	Ref.1
Fshr	TGCTCTAACAGGGTCTTCCTC	TCTCAGTTCAATGGCGTTCCG	242397445c1
Lhcgr	CGCCCGACTATCTCTCACCTA	GACAGATTGAGGAGGTTGTCAAA	7305233a1
Sox9	AGTACCCGCATCTGCACAAC	ACGAAGGGTCTCTTCTCGCT	165932320c1
Dmrt1	CAGAGGGACGCATGGTCATC	TGTAGTAGGCGGGGTCTGATA	158631231c1
Foxl2	AACACCGGAGAAACCAGACC	CGTAGAACGGGAACTTGGCT	designed using NCBI software
HPRT1	GCCCTGGCGTCGTGATTAGT	TGTCCTGACTTGCAGAACGAGC	designed using NCBI software
HSD3B2	ATCTAAGTTACGCCCTCTTC	CTTCTCTTCCTTGACTTCCT	KiCq Start Primers, Sigma-Aldrich
STAR	GACAAAGTGATGAGTAAAGTGG	GTAAGTAATGAGTGCTCGAC	KiCq Start Primers, Sigma-Aldrich
CYP17A1	GAAGTTATCATCAATCTGTGGG	GTCATTCGATAAACGGGAAG	KiCq Start Primers, Sigma-Aldrich
CYP19A1	ATGTGGACGTGTTGACCCTTCT	AACTACGTACCGTTCGAGAGGA	KiCq Start Primers, Sigma-Aldrich

References

1. Bouma, G.J., Hart, G.T., Washburn, L.L., Recknagel, A.K. & Eicher, E.M. Using real time RT-PCR analysis to determine multiple gene expression patterns during XX and XY mouse fetal gonad development. *Gene Expr. Patterns* **5,** 141-9 (2004).

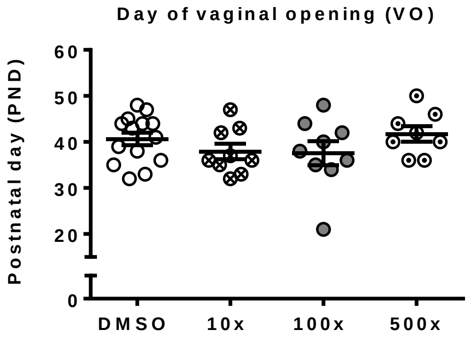

Figure S1. Day of vaginal opening (VO) for female offspring exposed *in utero* to DMSO (n=14) or to 10x (n=9), 100x (n=9) and 500x (n=8) the geometric mean of SELMA mothers' levels for the chemicals in Mixture S. Circles represent individual values, also means ± SEM are given. No significant differences were detected *vs*. DMSO.

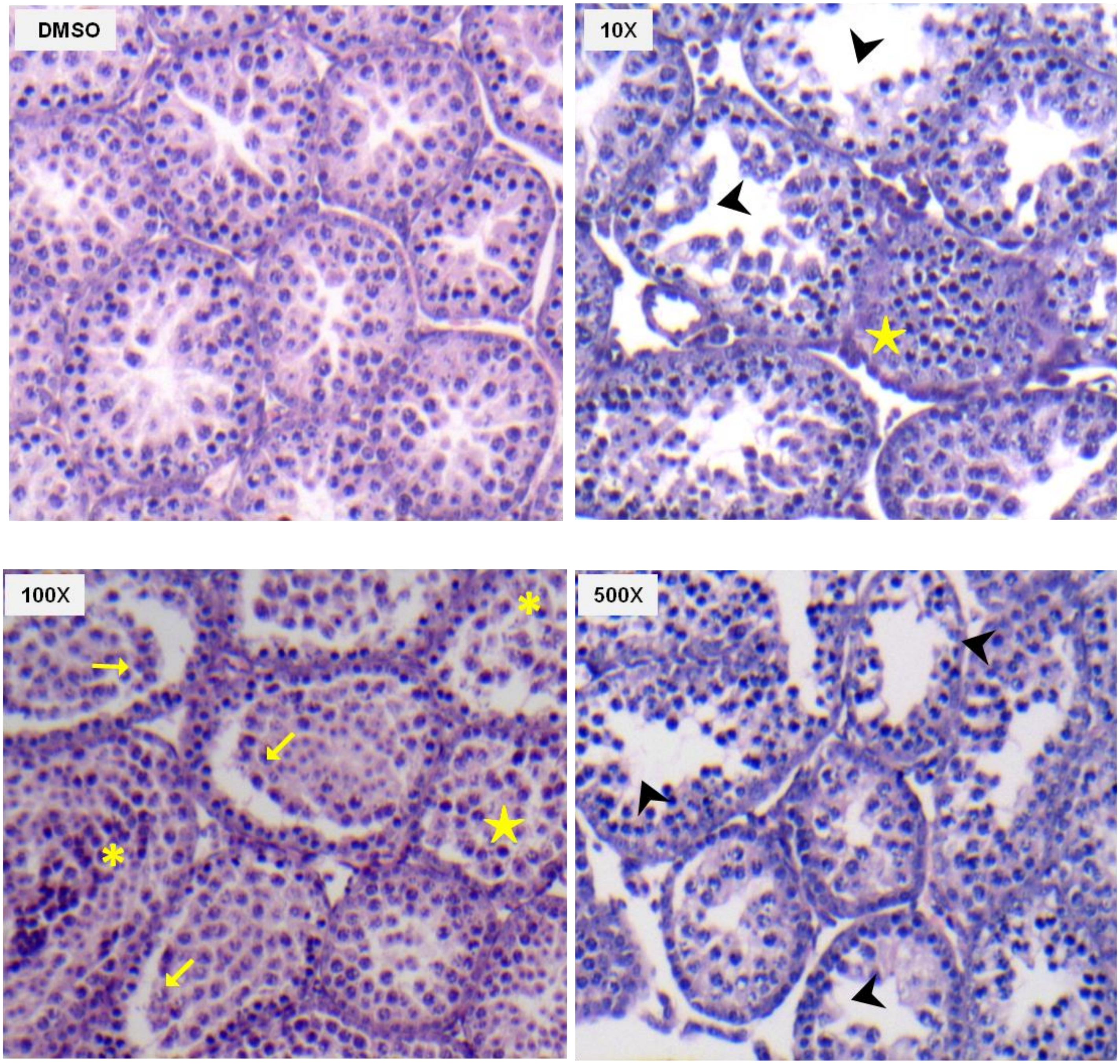

Figure S2A. Supplementary photomicrographs of testicular histology of PND21 offspring exposed to 0x (DMSO), 10x, 100x and 500x SELMA mothers' levels. ⋆ indicate tubules without lumen; * Indicate tubules with germ cells in the lumen; arrows indicate detached layers; arrowheads indicate tubules with abnormal layers.

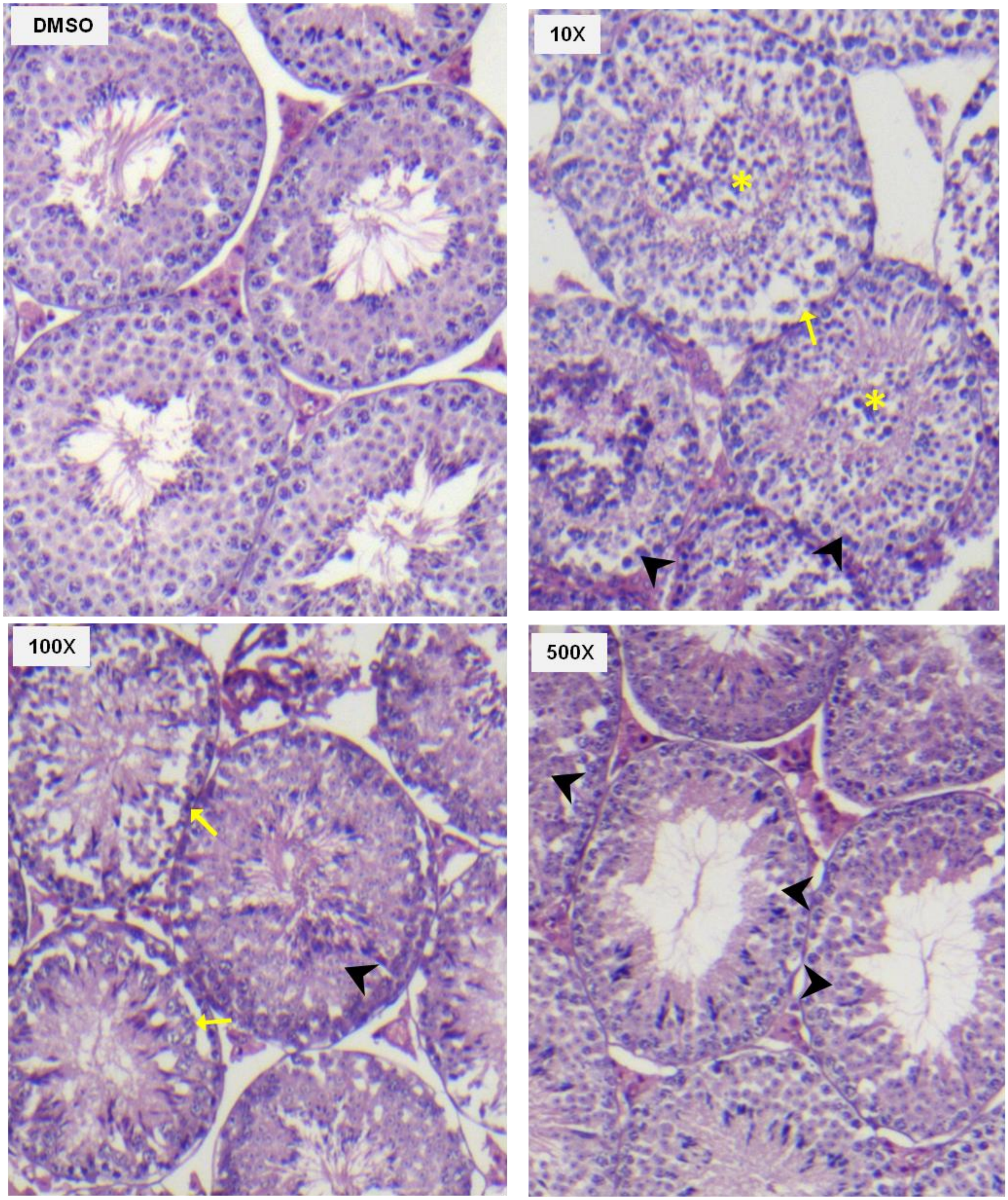

Figure S2B. Supplementary photomicrographs of testicular histology of PND90 offspring exposed to 0x (DMSO), 10x, 100x and 500x SELMA mothers' levels. * Indicate tubules with germ cells in the lumen; arrows indicate detached layers; arrowheads indicate tubules with abnormal layers.

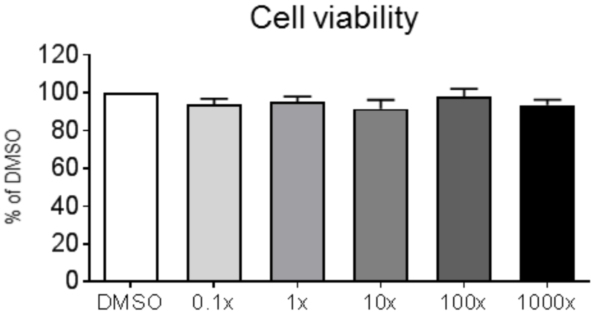

Figure S3. Cytoxicity assessment of Mixture S on H295R cells after 48 h of Mixture exposure. N = 3 experiments with 3 technical replicates; data represent mean ± SEM.

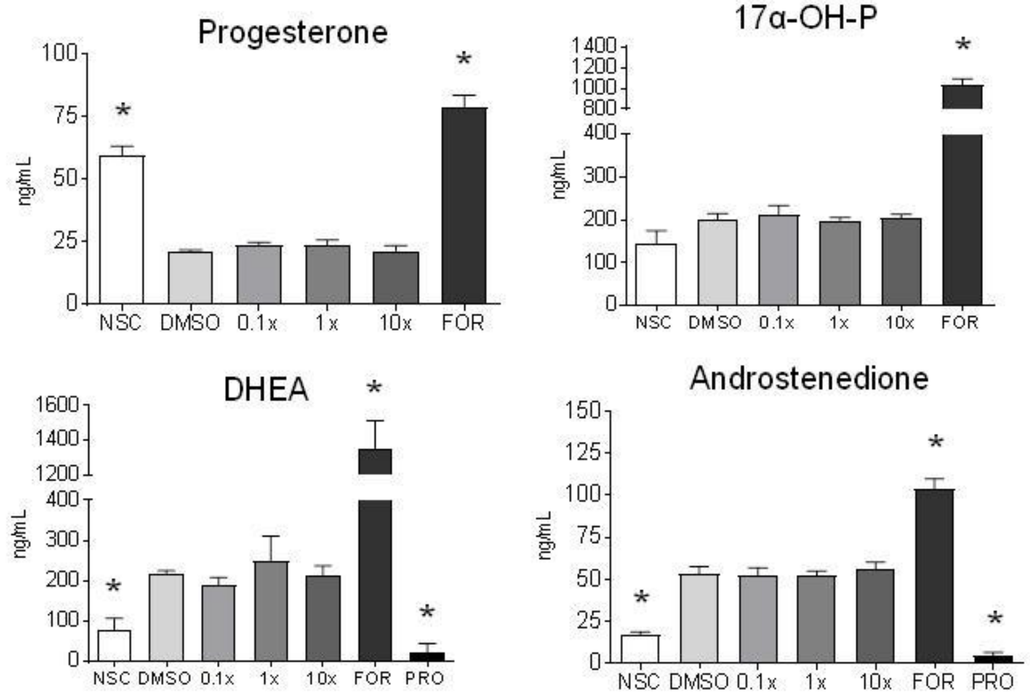

Figure S4. Production of steroid hormones by H295R cells after 24 h Mixture S exposure.NSC – non-stimulated, non-treated control; FOR – forskolin; PRO – Prochloraz. N = 3 experiments with 2 technical replicates; data represent mean ± SEM; * $p< 0.05$ vs. DMSO control; One-Way ANOVA with Dunnett's multiple comparison test.
